# SPP1 May Play an Important Role in the Carcinoid Nature of PAH

**DOI:** 10.1155/mi/7457106

**Published:** 2025-12-05

**Authors:** Yuxia Huang, Sheng Yan, Jing Zhu, Wentian Zhang

**Affiliations:** ^1^ Department of Pulmonary Diseases, Institute of Respiratory Diseases of Sun Yat-sen University, The Third Affiliated Hospital of Sun Yat-sen University, Guangzhou, China, zssy.com.cn; ^2^ Department of Operating Room, Shanghai Pulmonary Hospital, Tongji University School of Medicine, Shanghai, China, tongji.edu.cn; ^3^ Department of Thoracic Surgery, Shanghai Pulmonary Hospital, Tongji University School of Medicine, Shanghai, China, tongji.edu.cn

**Keywords:** gene expression, immune environment, nonsmall cell lung cancer, pulmonary arterial hypertension, single cell analysis

## Abstract

**Objection:**

Pulmonary arterial hypertension (PAH) was a cancer‐like disease. It shared several mechanisms, including perivascular inflammation. But the genes common and different between cancer and PAH was still unclear. We aimed to analyze the genes common in the two diseases, especially the N7‐methylguanosine (m7G) genes.

**Methods:**

We acquired dataset GSE1519, GSE113439, and GSE81089 and recognized differentially expressed genes (DEGs) and investigated their functions utilizing R software. m7G‐related genes were identified using a online tool RMvar. The extent of immune cell infiltration in the normal and PAH tissues, nonsmall cell lung cancer (NSCLC) tissues was determined using ImmuneCellAI and CIBERSORT. Additionally, the association between diagnostic markers and immune cells was analyzed. Single cell analysis and Cellchat were used to analyze the role of SPP1 in the PAH.

**Results:**

Among five DEGs overlapped by the differently datasets about NSCLC, CD163, and SPP1 were m7G genes. The immune cell infiltration results suggested that PAH and NSCLC shared different immune cell infiltration. SPP1 was significantly correlated to the macrophage cells and activated mast cells in NSCLC. Higher expression of CD163 and SPP1 might be related to the progression of monocrotaline (MCT) induced rats in the dataset GSE229361. The KM survival analysis suggested that higher expression of CD163 and SPP1 related to poor prognosis of NSCLC. The important role of SPP1 in PAH was verified using single cell anslysis.

**Conclusion:**

Different T cells infiltration contributed to the development of PAH and NSCLC. SPP1 might be vital for the cancer‐like characteristics of PAH.

## 1. Introduction

Pulmonary arterial hypertension (PAH) is a complex and progressive cardiopulmonary disorder, which was characterized by the pulmonary vascular remodeling [1]. Pulmonary vascular remodeling mostly occurred in the small to mid‐sized pulmonary arterioles (500 mm), is a feature of PAH [2]. In recent years, the novel cancer‐like concept for PAH has emerged and has its roots in intriguing in situ and in vitro observations [3, 4].

The dysfunctional immune system was present in nonsmall cell lung cancer (NSCLC) patients and played vital roles in the development of NSCLC [5]. The chronic inflammation in NSCLC changes the immune cells subtype and the function of immune cells are impaired in the microenvironment of the NSCLC. The increasing infiltration of immunosuppressing cells were related to the patient poor outcome [6]. Perivascular inflammation is the main characteristic of the PAH [7]. Like NSCLC, the infiltration of immune cells also exists in the PAH. It was reported that compared with control group, PAH patients had higher infiltration of natural killer (NK) cell activation, monocyte, T cell CD4 memory activation, and mast cell [8]. Various immune cells, including NK cells, cytotoxic T, NK, and NK T cells may contribute to vascular remodeling in the development of the PAH [9]. Deficiency of cytotoxic T cells is one of the poor prognostic factors in pulmonary hypertension [10]. Besides, higher expression of CD4+T cells were found to be one of the main infiltrating inflammatory cells [11]. The M2 macrophage was increased in the perivascular. M2 macrophages could promote pulmonary fibrosis by producing TNF‐α, IL‐1β, and so on [12]. Multiple studies proved that PAH and cancer shared several etiologies, including infiltration of immune and inflammatory cells, including macrophages, sustaining proliferative signaling, and resisting cell death [13, 14]. Like PAH, M2 macrophages played vital roles in the NSCLC. Tumor associated macrophages displayed M2 macrophages and were key component of tumor microenvironment. The infiltration of M2 macrophages was associated with the development of the NSCLC [15]. Besides, the immune system abnormality both exist in the PAH and NSCLC [16]. Furthermore, the properties of PAH were found in some NSCLC animal models, including vascular remodeling and perivascular inflammatory cell accumulation [17].

RNA methylation is a common epigenetic modification, which is a common characteristic of lung cancer and PAH [18, 19]. N7‐methylguanosine (m7G) methylation, the RNA methylation of guanine at position N7, and it is present in in all three transcript segments of mRNA 5′UTR, CDS, and 3′UTR and in pre‐mRNAs [20]. m7G methylation is a common mRNA cap modification at the 5′cap of eukaryotic mRNA, regulating mRNA function, including mRNA export, translation, and splicing [21]. The modification of m7G was related to various disease and these changes had played vital role in the development of many disease, especially cancer [22].

mRNA methylation played vital roles in the immune response, but the role of the RNA methylation in the immune regulation of PAH is still unclear. We try to identify the relationship of the m7G‐related genes in the immune infiltration in the NSCLC and PAH and found out their similarity.

## 2. Methods

### 2.1. Data Acquisition

We downloaded the GSE15197 dataset (31), the GSE113439 dataset (32), and the GSE81089 from the Gene Expression Omnibus (GEO) database. The dataset GSE15197 included lung tissue samples from 26 patients with PAH and 13 normal, including PAH subjects (*n* = 18), IPF subjects with secondary PH (*n* = 8), and normal (*n* = 13). The GSE113439 dataset included lung tissue from 14 patients with PAH and 11 normal, of which the PAH group included six patients with idiopathic PAH, four patients with PAH secondary to connective tissue disease (CTD), and four patients patients with chronic heart disease (CHD). The GSE81089 contains RNA seq from fresh tissues of 199 NSCLC patients and paired para‐tumor tissues. To identify the common gene distribution between the NSCLC and PAH patients and in order to achieve a larger sample size, we integrated the GSE113439 and GSE15197, raw counts were processed using DESeq2. Data were log2 transformed to approximate normal distribution. Batch correction was performed using ComBat, and residual batch effects were examined using PCA. To validate the common genes of the datasets, we used GSE117261 and GSE81096 for validation. GSE117261 was a RNA‐seq dataset containing 58 PAH patients and 25 control lung tissues, GSE81096 was a RNA‐seq dataset containg fresh frozen tumor tissues from 199 patients diagnosed with NSCLC and 19 paired normal lung tissues.

### 2.2. Identify the Differentially Expressed Genes (DEGs)

To identify the common DEGs between the PAH and NSCLC patients, genes expressed differently between the PAH and control group or NSCLC and control group were identified using DESeq R package (1.18.0), respectively, and then screened by the Venn. The *p* values were adjusted using the Benjamini and Hochberg method. The *p* value of 0.05 and log2 (fold change) of 1 were set as the thresholds for significantly differential expression.

### 2.3. Functional Enrichment of the DEGs in PAH and Lung Cancer

Gene ontology (GO) and Kyoto Encyclopedia of Genes and Genomes (KEGG) enrichment analysis were performed by an online software DAVID (https://david.ncifcrf.gov/) version 6.8. *p* value < 0.05 was considered to indicate a statistically significant difference. We performed GO and KEGG analysis for the DEGs of PAH and NSCLC respectively.

### 2.4. Immune‐Related Analysis of the m7G‐Related Genes Among the PAH and NSCLC Patients

In order to identify the role of m7G‐related genes among the PAH and NSCLC patients, we screened and identified the m7G‐related genes using the online database RMvar (http://rmvar.renlab.org). Next we studied the correlation between m7G‐related genes and immune infiltration. The infiltration score calculations were made using CIBERSORT and ImmuCellAI. ESTIMATE algorithm was applied to show the presence of infiltrated immune cells in the PAH and NSCLC, respectively.

To identify the key immune signals, the predefined immune‐related gene set (c7.immunesigdb.v7.5.1.symbols.gmt) was downloaded from the Molecular Signatures Database (MSigDB) (http://www.gsea-msigdb.org/gsea/msigdb/). Based on the R package GSEA (version 4.1.0), and the functional enrichment analysis of the genes profile was subsequently performed on the predefined gene set. False discovery rate (FDR) < 25% and nom *p* value < 0.05 were set as the cut‐off criteria.

### 2.5. Evaluation of m7G‐Related Genes and the Progression of the Monocrotaline (MCT)‐Induced Rats

We selected the dataset (GSE229361) to validate the relationship between the gene expression of the CD163, SPP1, and the progression of MCT‐induced rats through constructing the heatmap. GSE229361 was a RNA sequencing analysis of MCT induced PAH rats.

### 2.6. Survival Analysis

We selected GSE68465 for validation of the prognostic value of CD163 andSPP1.

For patients with NSCLC who have different treatment options, this study selected GSE135222 and GSE207422. The former is an RNAseq data of 27 NSCLC patients receiving immunotherapy, and the latter is an RNAseq data of NSCLC with different responses to immunotherapy.

Match probes to gene symbols using the annotation files provided by the manufacturer. If multiple probes matched to a single gene, we used the median rank value to calculate the expression value. Normalize and log2 transform expression data for further analysis. Here, each possible cutoff between the lower and upper quartiles is examined and the FDR is calculated using the Benjamini–Hochberg method to correct for multiple hypothesis testing. Survival analyzes were performed on recurrence‐free survival (RFS). With the same *p*‐value, the strongest hazard ratio was identified. Export the best cutoff results for each gene into a separate database, these results are used to generate Kaplan–Meier plots.

### 2.7. Single Cell Studies Reveal that SPP1 Plays an Important Role in the Progression of PAH

For single cell data mining analysis, we searched the GEO and Expressarray databases using the keywords “Pulmonary arterial hypertension” and “single cell.” We downloaded the single cell data GSE210248, including the original sequencing data, the aligned single cell expression matrix, and cell annotation information, and used the R studio software package Seurat to process and analyze the data. Cell type identification and screening: use clustering or dimensionality reduction methods to identify and classify the single cell expression matrix, and then filter it; cell–cell communication network construction: by calculating the gene expression similarity between different cell types, receptor pairing, and other different indicators, different cell–cell communication networks are established using CellChat.

### 2.8. Single Cell Studies Reveal that the Role of SPP1 in the Progression of NSCLC

For single cell data mining analysis, we searched the GEO and Expressarray databases using the keywords “nonsmall cell lung cancer” and “single cell.” We downloaded the single cell data GSE131907, including the original sequencing data, the aligned single cell expression matrix, and cell annotation information, and used the R studio software package Seurat to process and analyze the data. Cell type identification and screening: use clustering or dimensionality reduction methods to identify and classify the single cell expression matrix, and then filter it.

### 2.9. Statistical Analysis

All statistical analyses were performed using SPSS 26.0 (Statistical Package for the Social Sciences, Chicago, IL, USA), RStudio V1.2.1335 (RStudio Inc., Boston, MA, USA), and GraphPad Prism (San Diego, CA, USA) version 9.0. Continuous variables were represented by the mean, standard deviation (SD), or medians (interquartile range), whereas categorical variables were represented by percentage numbers (percent). When comparing clinical characteristics of patients, Chi‐square tests were employed to analyze categorical data, while the Student’s *t*‐test or Mann–Whitney *U* test was used to analyze continuous data.

For the RNA seq dataset, FPKM values were quantile normalized, log2 transformed, median centered, and the samples were split in two groups according to classification.

ImmuneCellAI algorithm and CIBERSORT algorithm were used to estimate the presence of different immune cells in tumor and ESTIMATE algorithm was used to calculate immune score for the level of immune cells in PAH and tumor tissues.

## 3. Results

### 3.1. Identification of Different Expressed Genes in PAH and NSCLC Dataset

Based on the criteria of *p* value < 0.05 and |log2foldchange| > 1, there were 2523, 630, and 5316 DEGs in the dataset GSE15197, GSE113439, and GSE81089, respectively. We integrated the GSE15197 and GSE113439 into a new dataset. As shown in Figure 1, the dataset GSE15197 and GSE113439 were normalized and integrated. We identified 69 DEGs, after verified by the dataset GSE117261 and GSE116959, five genes including MS4A15, PI15, CD163, POSTN, and SPP1 were acquired.

Figure 1The different expressed genes between PAH and NSCLC. (A) Before the sample normalizatd; (B) after the sample normalizatd; (C) identify the common differently expressed genes between PAH and NSCLC.

**Figure:**
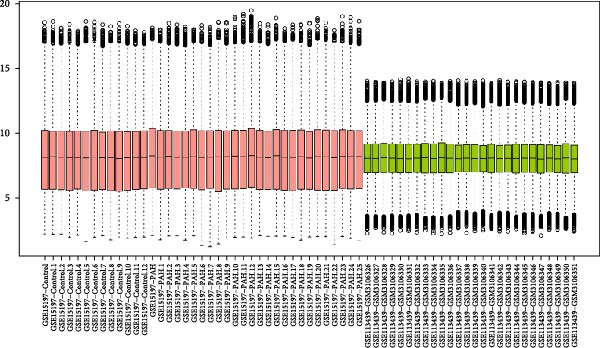
(A)

**Figure:**
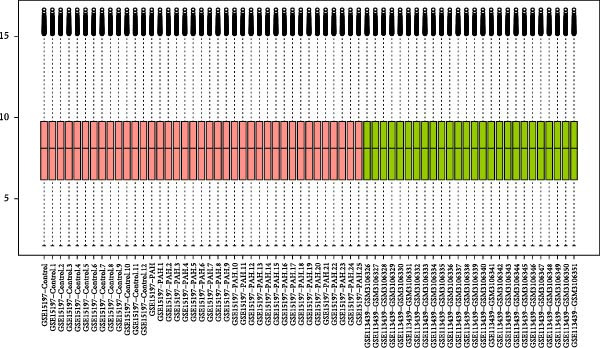
(B)

**Figure:**
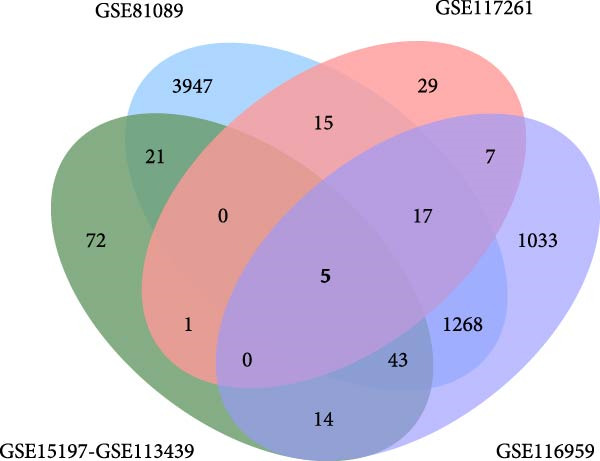
(C)

### 3.2. GO and KEGG Enrichment of the Different Expressed Genes in PAH and NSCLC

As shown in Figure 2, the GO analysis of the DEGs in the GSE15197 and GSE113439 integrated dataset suggested that the DEGs were mainly enriched in the response to endogenous stimulus, extracellular region part, extracellular region, inflammatory response, and regulation of response to external stimulus. KEGG analysis suggested that the DEGs were enriched in the neuroactive ligand–receptor interaction, lipid and atherosclerosis, African trypanosomiasis, and interleukin‐17 (IL‐17) signaling pathway.

Figure 2GO and KEGG analysis of DEGs between the PAH and NSCLC. (A) GO analysis of differently expressed genes of PAH; (B) KEGG analysis of differently expressed genes in PAH; (C) GO analysis of differently expressed genes in NSCLC; (D) KEGG analysis of differently expressed genes in NSCLC.

**Figure:**
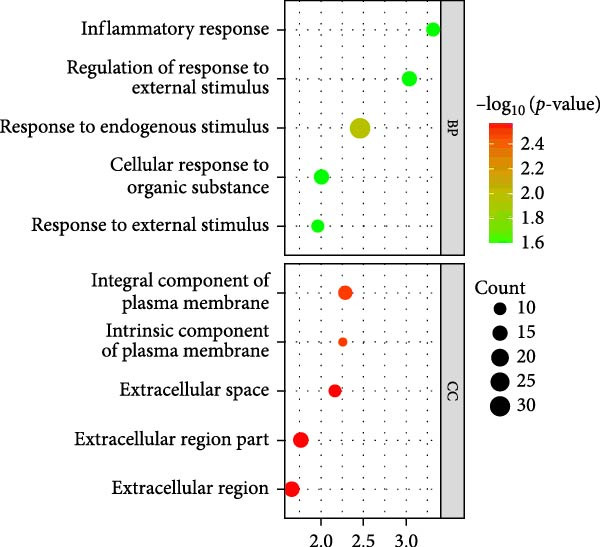
(A)

**Figure:**
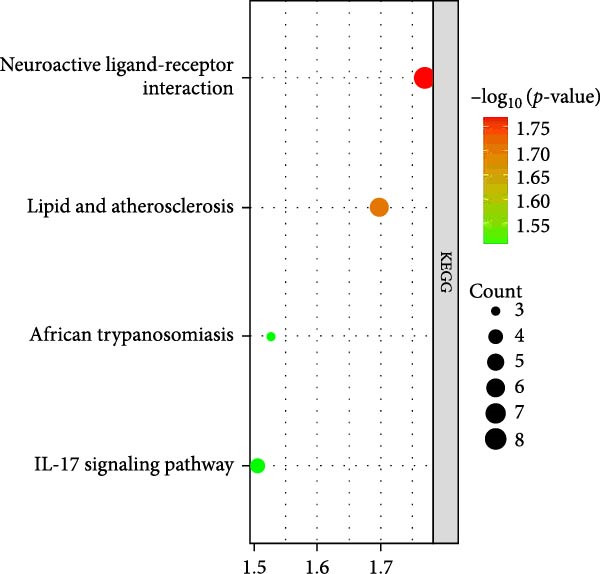
(B)

**Figure:**
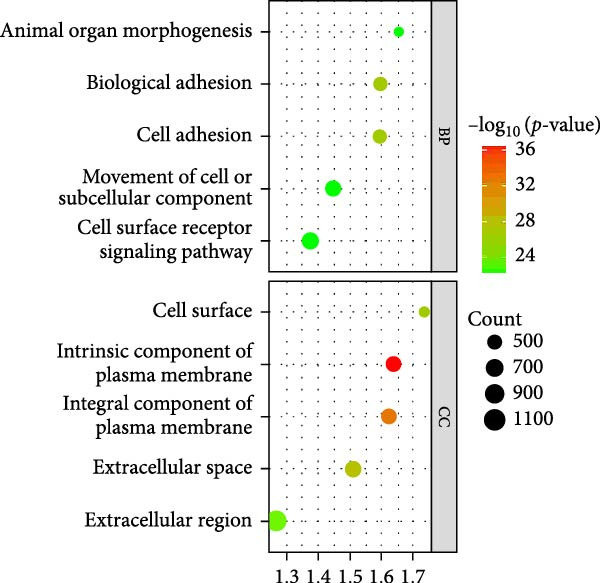
(C)

**Figure:**
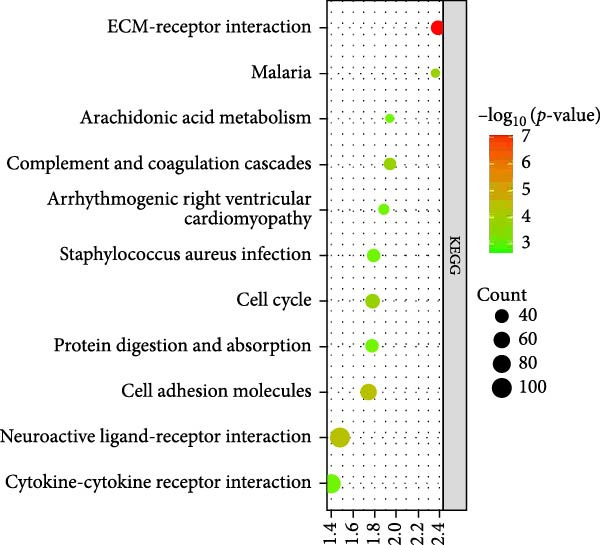
(D)

As for the GSE81089 dataset, the DEGs were enriched in the intrinsic component of plasma membrane, integral component of plasma membrane, extracellular space, cell surface, and biological adhesion. KEGG analysis suggested that the DEGs were mainly related to ECM–receptor interaction, neuroactive ligand–receptor interaction, cell adhesion molecules, malaria, and cell cycle.

### 3.3. Analysis of the Immune Cells Infiltration in the PAH and NSCLC Patients

Among the five overlapped DEGs, CD163 and SPP1 were found be m7G‐related genes.

As shown in Figure 3, the ImmuneCellAI analysis showed that B cell, monocyte, gammadelta T cells, Tr1 cells, nTreg cells, Th2, Th17, CD8+ naïve T cells, and effecter memory cells were different in the PAH and control group. DC cells, monocytes, neutrophil cells, gammadelta T cells, Tr1 cells, nTreg cells, iTreg cells, Th2 cells, Th17 cells, CD8+ naïve cells, cytotoxic cells, exhausted cells, and effector memory cells were elevated in the NSCLC group.

Figure 3Immunecell filtration analysis of DEGs through ImmuneCellAI. (A,B) Immunecell filtration of DEGs in PAH. (C,D) Immunecell filtration of DEGs in NSCLC.

**Figure:**
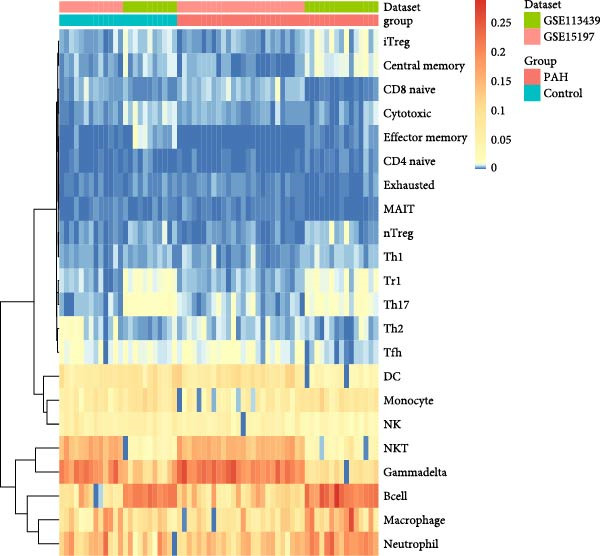
(A)

**Figure:**
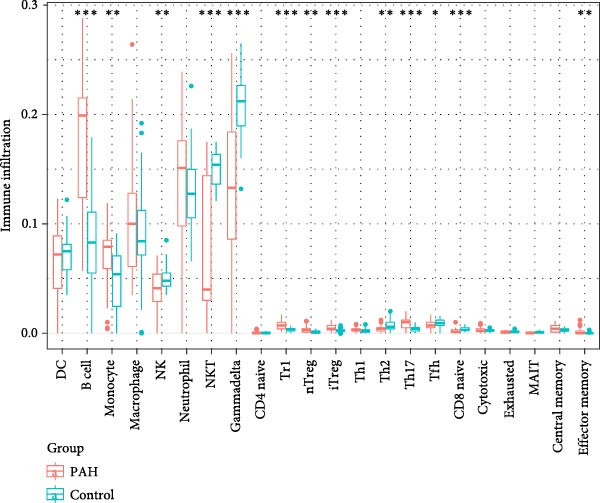
(B)

**Figure:**
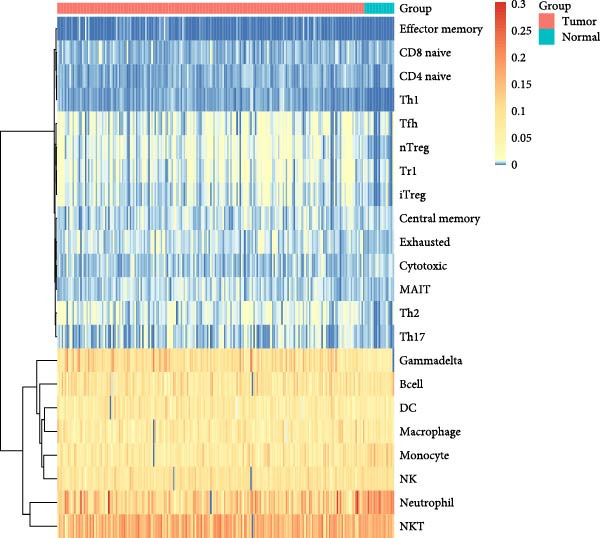
(C)

**Figure:**
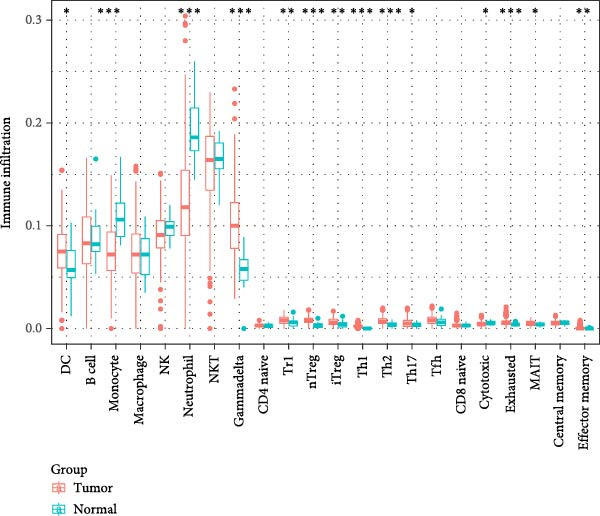
(D)

As shown in Figure 4, and according to the CIBERSORT algorithm, CD8+T cells, monocytes, resting mast cells, and activated mast cells were elevated in the PAH group. B cells, plasma cells, CD4+ memory resting T cells, CD4+ memory activated T cells, and M1 macrophage were elevated in the NSCLC group.

Figure 4Immunecell infiltration analysis of DEGs through CIBERSORT algorithm. (A,B) Immunecell filtration of DEGs in PAH. (C,D) Immunecell filtration of DEGs in NSCLC.

**Figure:**
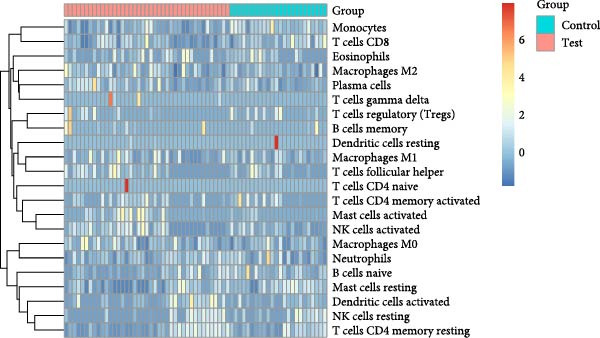
(A)

**Figure:**
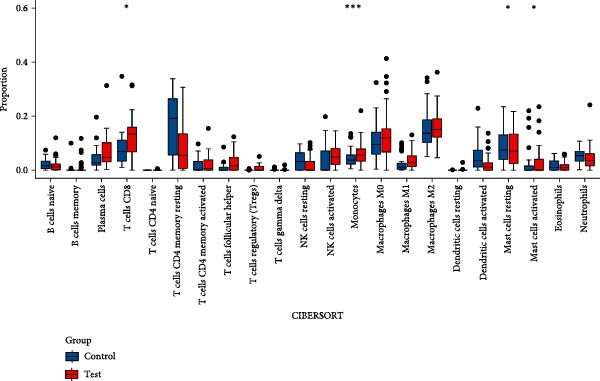
(B)

**Figure:**
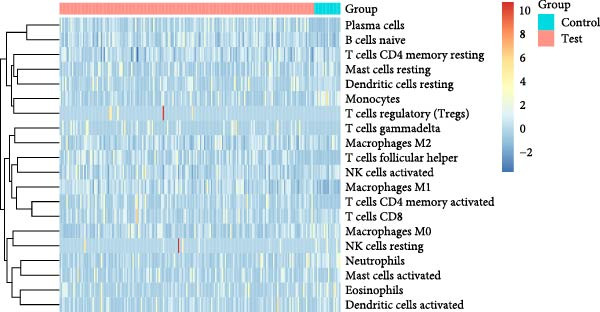
(C)

**Figure:**
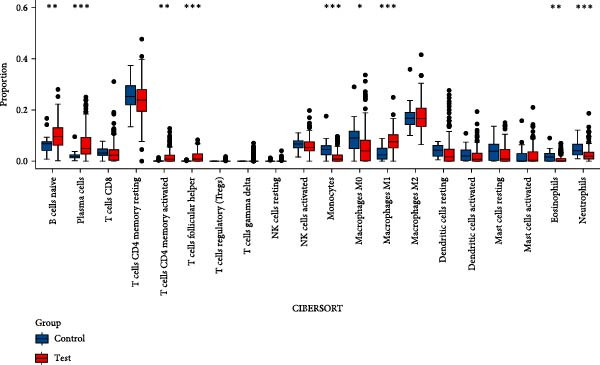
(D)

### 3.4. The Correlation Between the m7G‐Related Genes and the Immune Microenvironment in PAH and NSCLC

As shown in Figure 5, the expression of CD163 in PAH was significantly correlated to the several subtypes of T cells and NK cells, including T cells follicular helper, CD4+ memory T cells, and activated NK cells (*p*  < 0.05). SPP1 was significantly correlated to the M0 macrophage and nTreg cells (*p*  < 0.05).

Figure 5The relationship between the m7G related genes and the immune microenvironment in PAH. (A) The expression of CD163 in PAH was significantly correlated to the several subtypes of T cells and NK cells in PAH. (B) SPP1 was significantly correlated to the M0 macrophage and nTreg cells.

**Figure:**
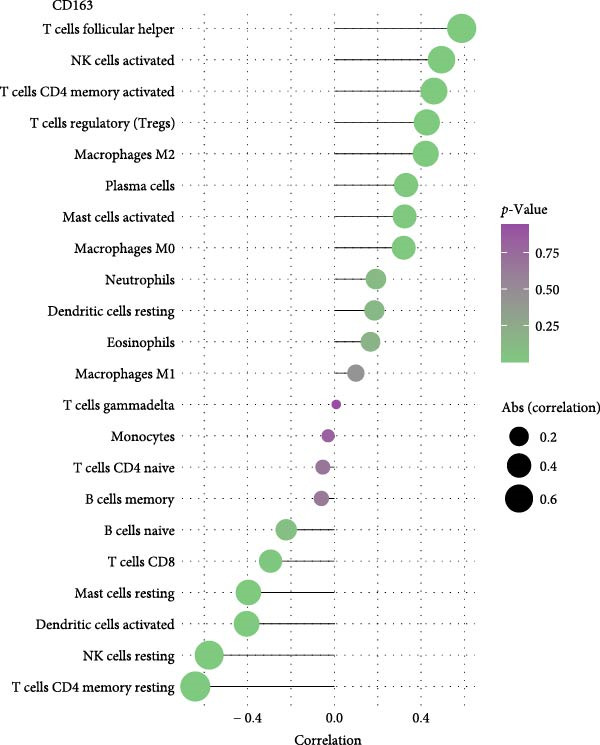
(A)

**Figure:**
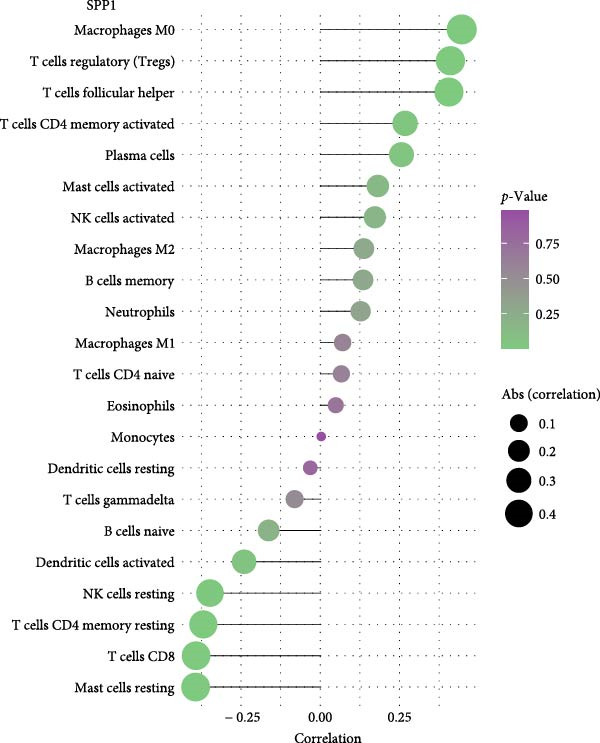
(B)

As for the NSCLC group (Figure 6), the expression of CD163 in NSCLC was significantly correlated to M2 macrophage cells, CD8+T cells, and activated CD4+memory T cells (*p*  < 0.05). SPP1 was significantly correlated to the M1 macrophage cells, M2 macrophage cells, and activated mast cells (*p*  < 0.05).

Figure 6The relationship between the m7G related genes and the immune microenvironment in NSCLC. (A) The expression of CD163 in NSCLC was significantly correlated to M2 macrophage cells and several types of T cells. (B) SPP1 was significantly correlated to the M1 macrophage cells, M2 macrophage cells, and activated mast cells.

**Figure:**
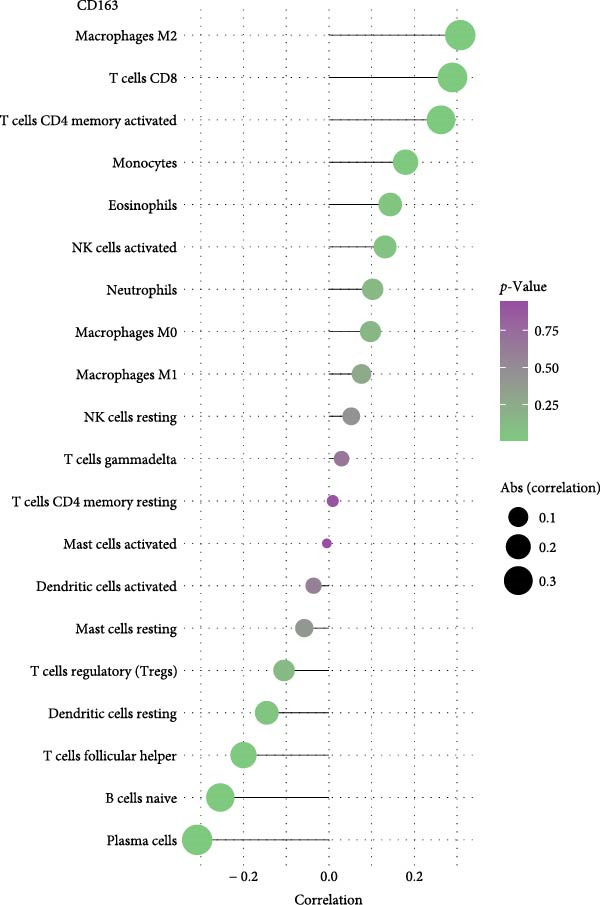
(A)

**Figure:**
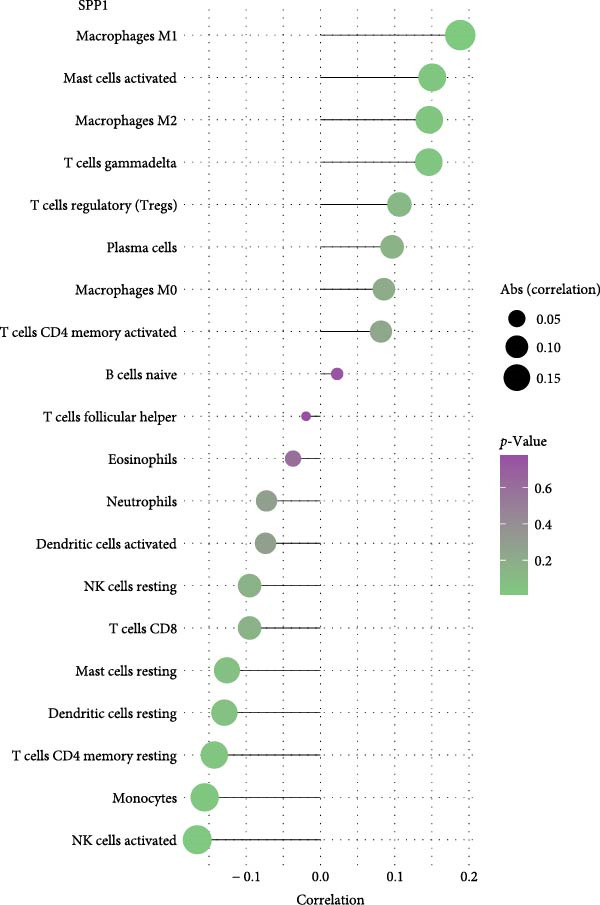
(B)

### 3.5. The Correlation Between the m7G‐Related Genes and the Disease Progression in MCT Rats

We analyzed the gene expression between rats treated with MCT for 1 week and 3 weeks in GSE229361. We found that compared with the rats treated with MCT for 1 week, CD163 and SPP1 were upregulated in the rats treated with MCT for 3 weeks (Figure 7).

Figure 7The relationship between the m7G related genes and the prognosis of PAH. (A) CD163 and SPP1 was upregulated in the MCT‐rat. (B) CD163 and SPP1 was differently expressed in the lung tissue of rat treated with MCT for 3 weeks.

**Figure:**
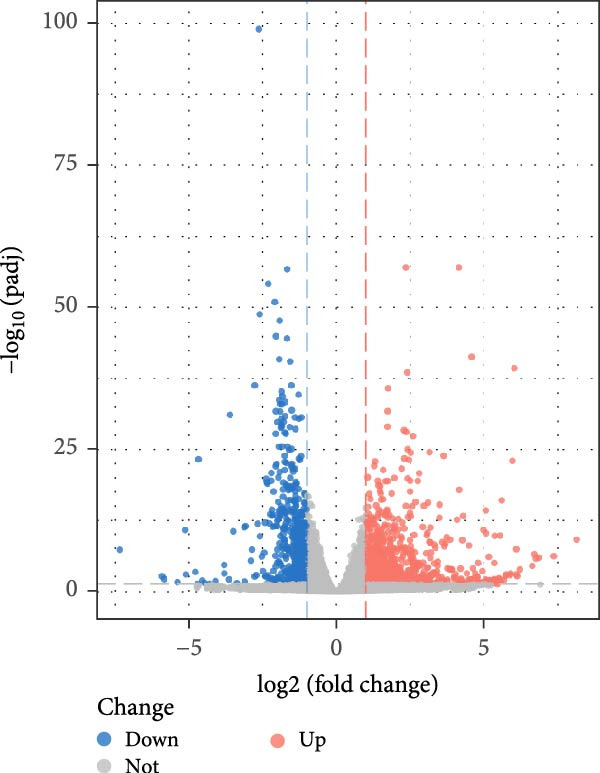
(A)

**Figure:**
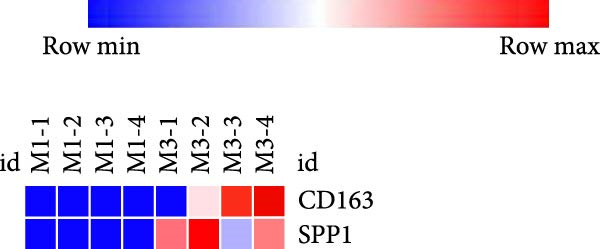
(B)

### 3.6. Validation of Prognostic Value of CD163 and SPP1 in NSCLC Group

The survival analysis of CD163 and SPP1 in NSCLC group using the KM plotter and demonstrated that the expression of CD163 and SPP1 were related to the prognosis of the NSCLC. The higher expression of CD163 or SPP1, had a worse prognosis (*p*  < 0.05) (Figure 8A,B). The ROC curve showed that there no significant statistical difference in the PFS with patients with different SPP1 expression levels (*p*  > 0.05) (Figure 8C), and there was no significant statistical difference in the expression of SPP1 between NSCLC patients with and without immune response (*p*  > 0.05) (Figure 8D,E).

Figure 8The prognosis prediction value of CD163 and SPP1 in NSCLC. (A) KM plotter suggested that the higher expression of CD163 had a worse prognosis. (B) KM plotter suggested that the higher expression of SPP1 had a worse prognosis. (C) The ROC curve showed that there was no significant statistical difference in the PFS between NSCLC patients with differently expression of SPP1. (D,E) No significant statistical difference was shown in the expression of SPP1 between NSCLC patients with and without immune response.

**Figure:**
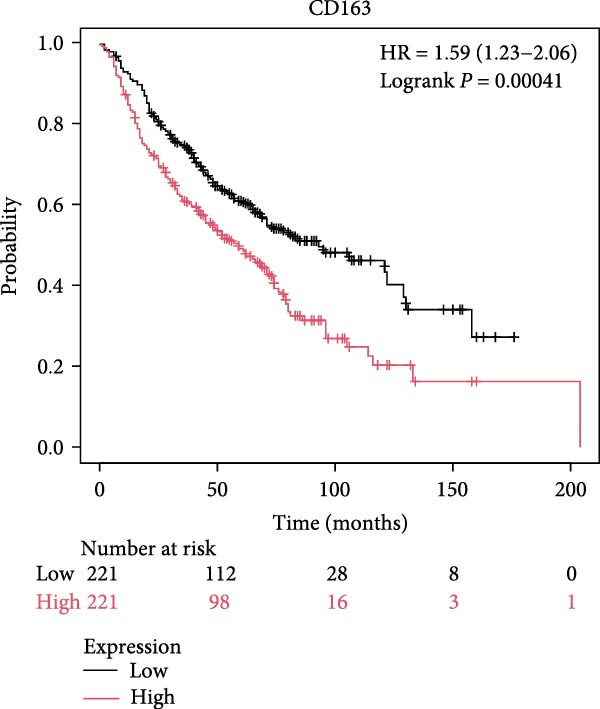
(A)

**Figure:**
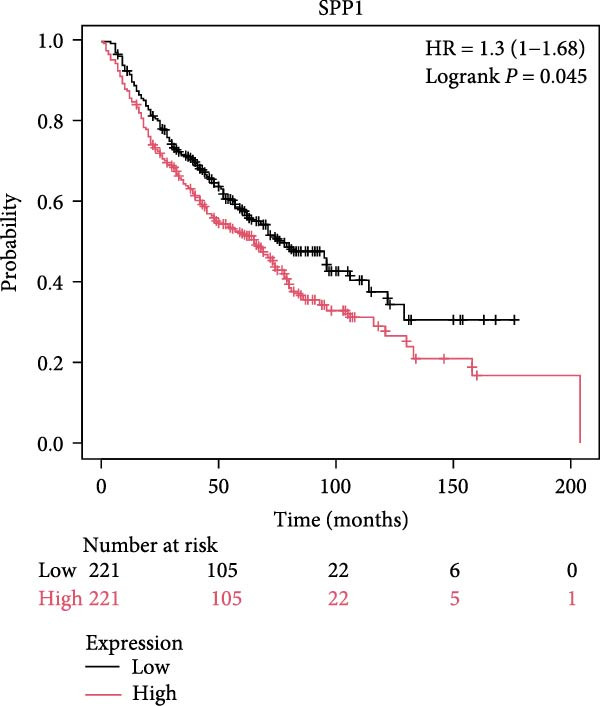
(B)

**Figure:**
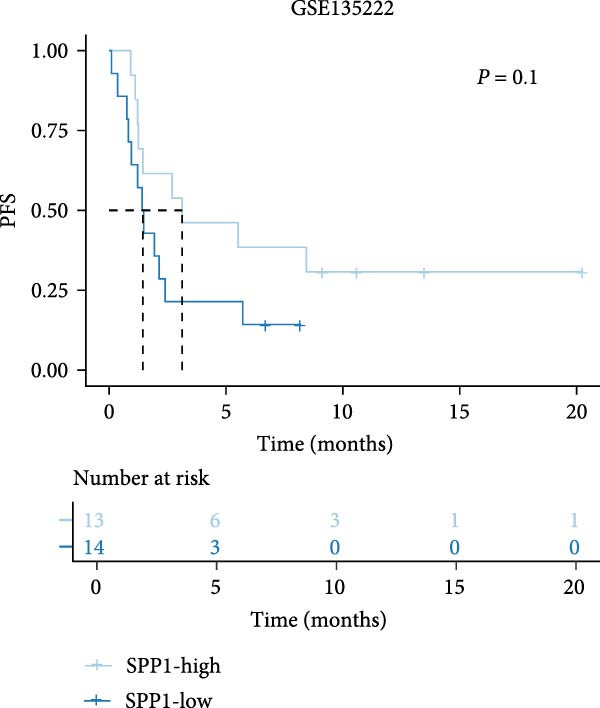
(C)

**Figure:**
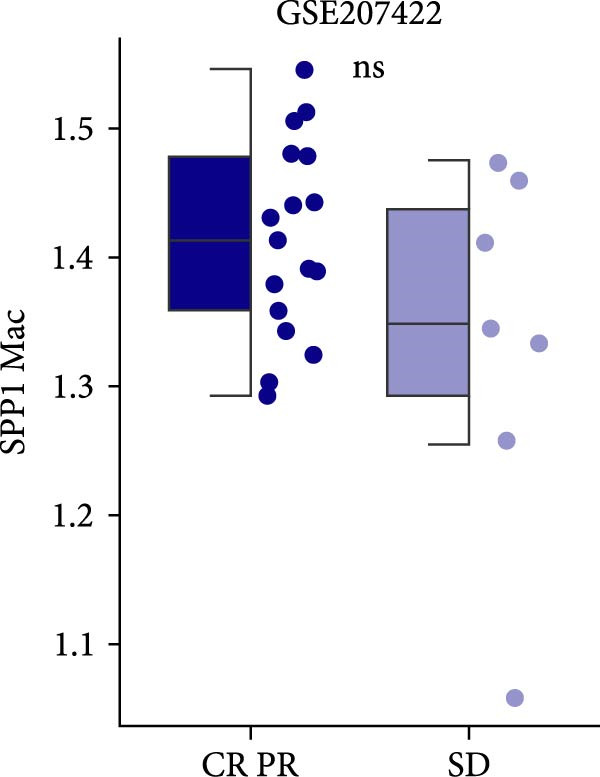
(D)

**Figure:**
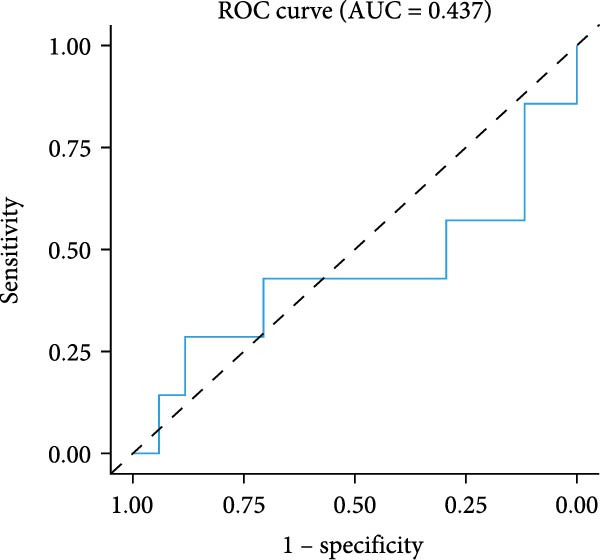
(E)

### 3.7. The Important Role of SPP1 Identified by the Single Cell Analysis

**Figure 10:**
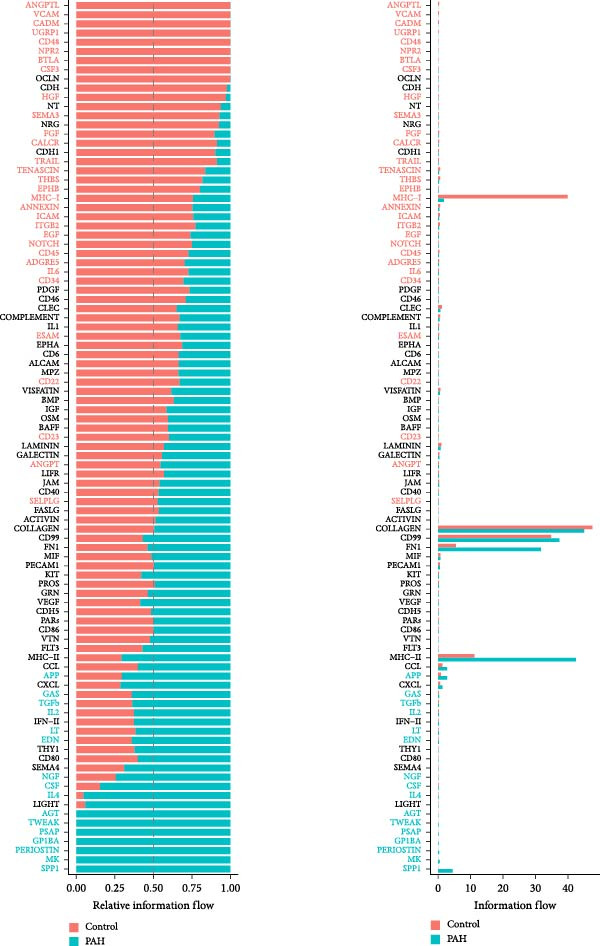
The significant pathways related to SPP1 in PAH. The comparison of signaling pathway based on the relative information flow between pairwise datasets showed that SPP1 related pathways played important roles in the PAH.

As shown in Figure 9, in the GSE210248 single cell expression matrix, it was divided into PAH group and transplant donor group, with three cases in each group. In the cell population clustering, we used the UAMP clustering method to classify the cells into epithelial cells, endothelial cells, fibroblasts, mast cells, myeloids, and T/NK cells. The cell specific markers found using the FindAllMarkers in the Seurat. Figure 9B shows that SPP1 is highly expressed in myeloid cells. Then we showed the all significant signaling pathways that were ranked according to their differences of overall information flow in Figure 10.

Figure 9The cell distribution of SPP1 in the PAH. (A) It was divided into PAH group and transplant donor group using Single Cell Analysis. (B) The differently distribution of SPP1 in the different cells of PAH.

**Figure:**
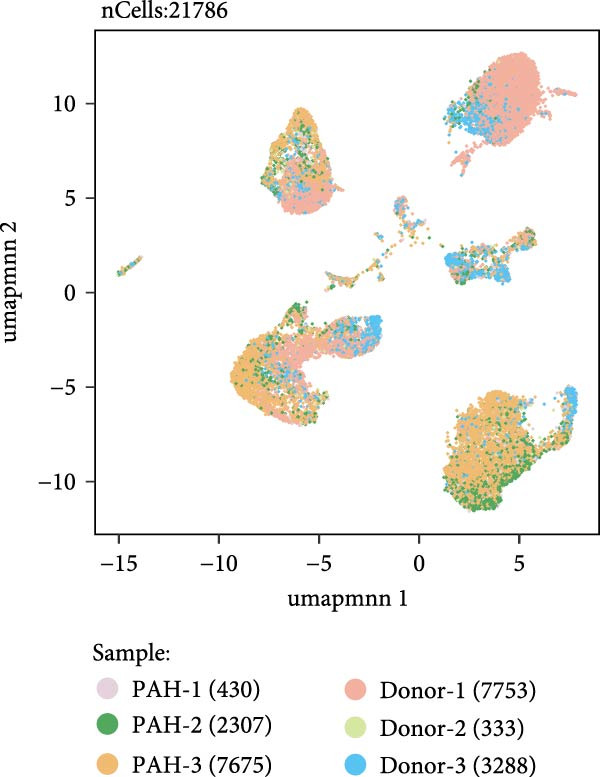
(A)

**Figure:**
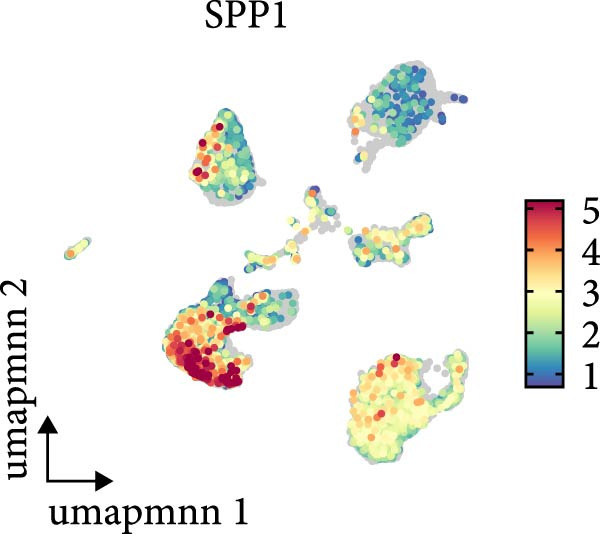
(B)

As shown in Figure 10, the information flow chart showed that SPP1 had a relatively higher information flow in PAH relative to control cells. Figure 11 shows that SPP1 relative signaling pathways were upregulated in the PAH, and SPP1 signaling network was participating in multiple cells.

Figure 11SPP1‐related pathways are important in many cells. (A) Networks of cell–cell inferred interactions show the number of SPP1‐(ITGAV+ITGB5) between different cells. (B) Interactions show the number of SPP1‐CD44 between different cells. (C) Interactions show the number of SPP1‐(ITGAV+ITGB1) between different cells. (D) Interactions show the number of SPP1‐(ITGAV+ITGB4) between different cells. (E) Interactions show the number of SPP1‐(ITGA8+ITGB1) between different cells. (F) Interactions show the number of SPP1‐(ITGA5+ITGB1) between different cells.

**Figure:**
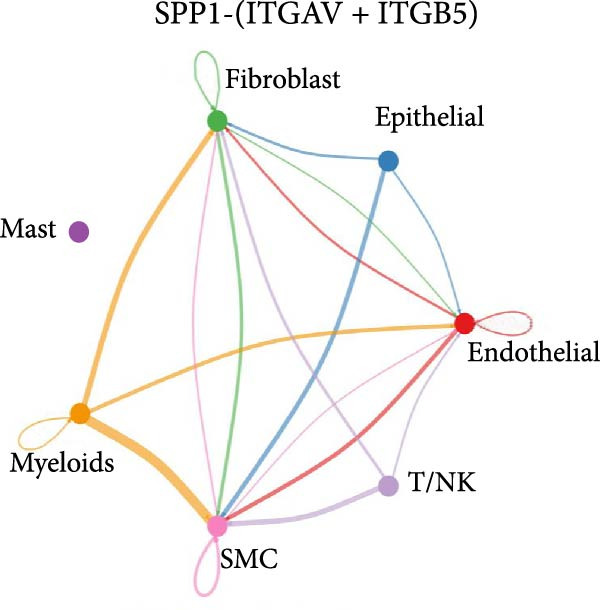
(A)

**Figure:**
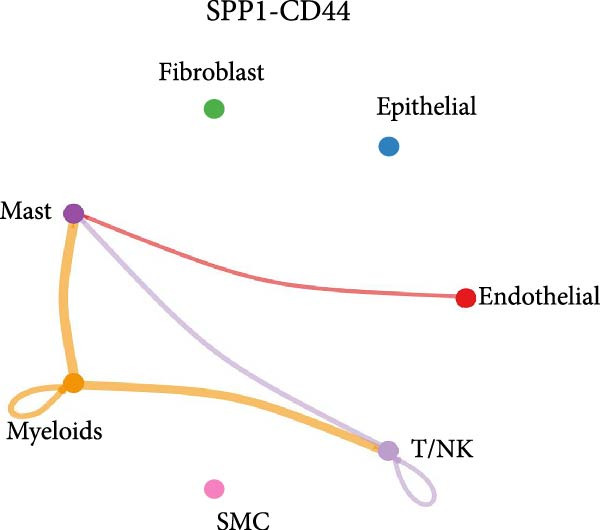
(B)

**Figure:**
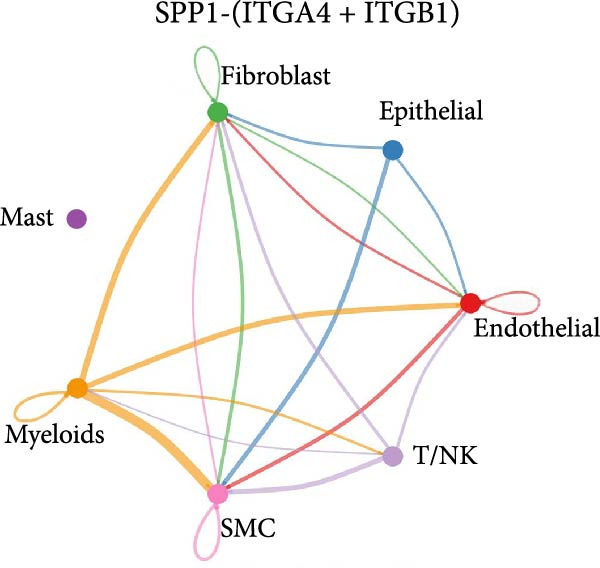
(C)

**Figure:**
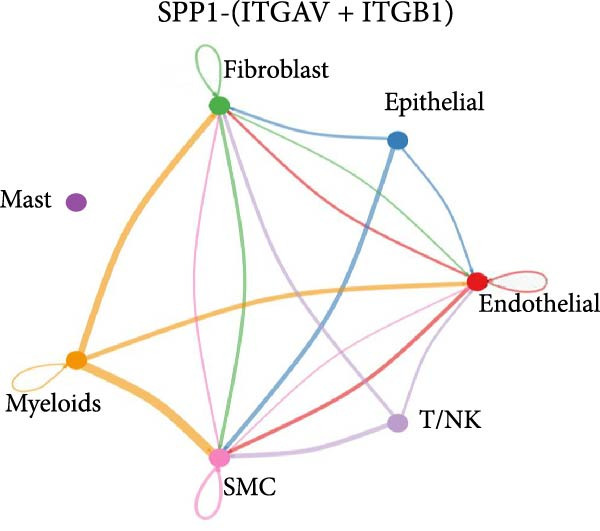
(D)

**Figure:**
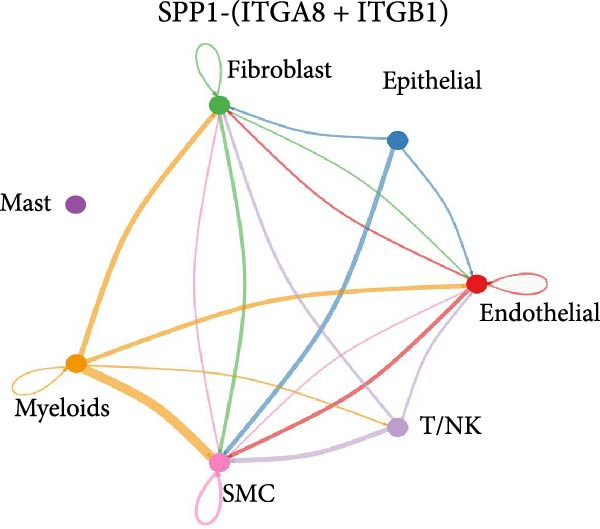
(E)

**Figure:**
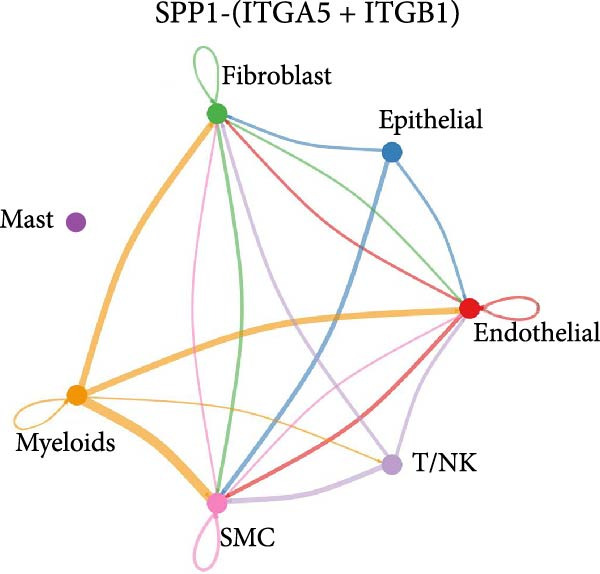
(F)

As shown in Figure 12A, in the GSE131907 single cell expression matrix, it consisted tumor tissue of 11 NSCLC patients. In the cell population clustering, we used the UAMP clustering method to classify the cells into epithelial cells, endothelial cells, fibroblasts, mast cells, myeloids, B cells, and T/NK cells. Figure 12B shows that SPP1 is highly expressed in myeloid cells. The cell specific markers found using the FindAllMarkers in the Seurat (Figure 12C).

Figure 12Cellular distribution of SPP1 in NSCLC. (A) We used the UAMP clustering method to classify the cells into epithelial cells, endothelial cells, fibroblasts, mast cells, myeloids, B cells, and T/NK cells in a NSCLC dataset. (B) SPP1 is highly expressed in myeloid cells. (C) Violin plots showing the expression of commonly used cell type marker genes across all clusters.

**Figure:**
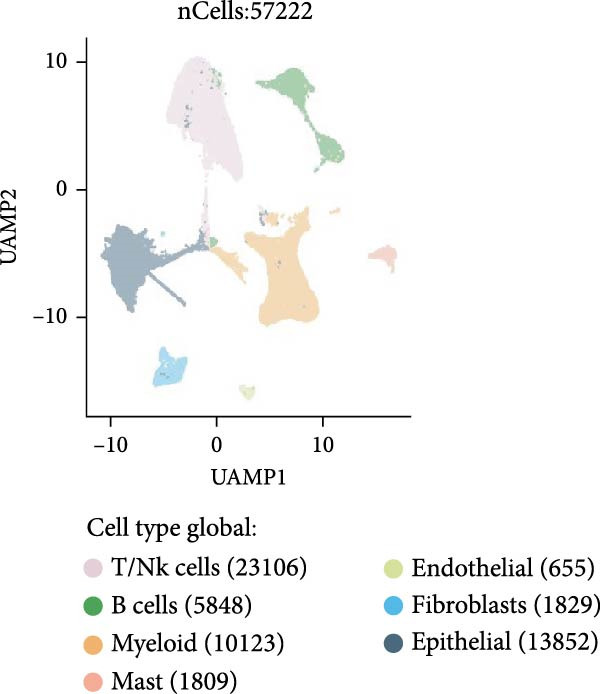
(A)

**Figure:**
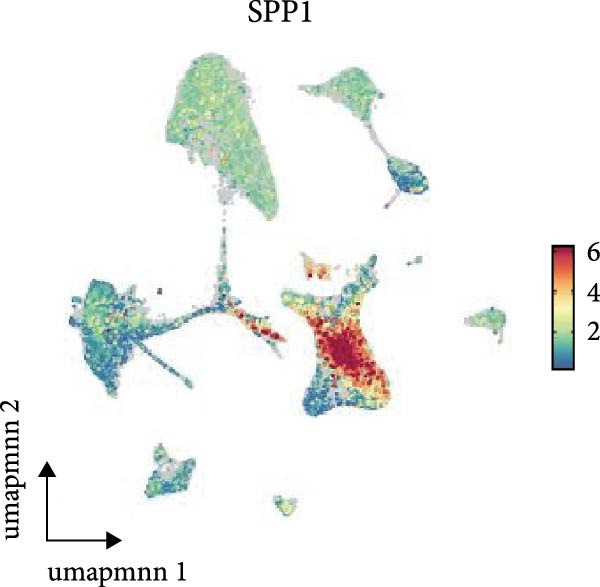
(B)

**Figure:**
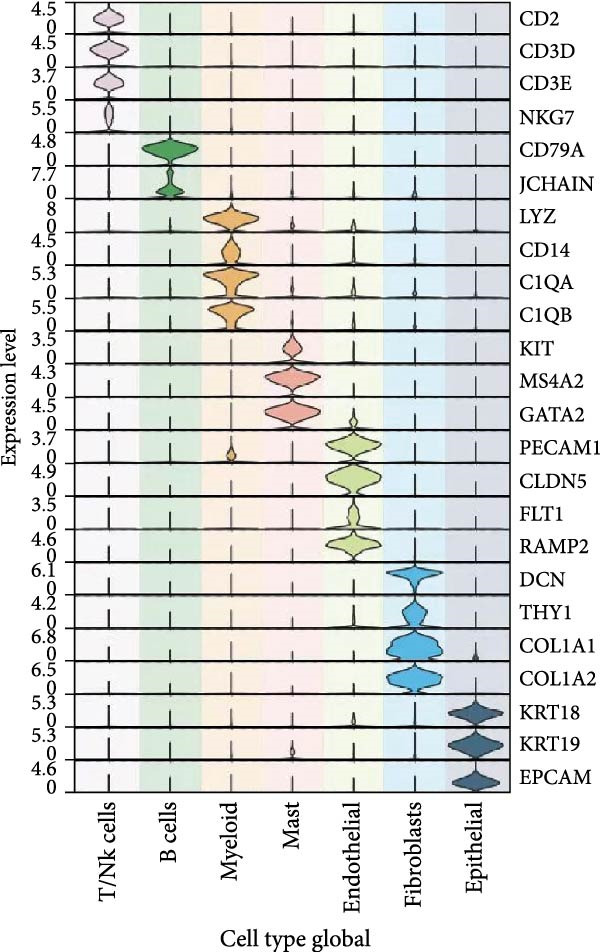
(C)

## 4. Discussion

PAH was a cancer‐like disease. The increased proliferation and reduced apoptosis of the pulmonary vascular vessels contributed to the development of the PAH. In our studies, we compared the immune infiltration between the PAH and NSCLC. The T cells infiltrations contributed to the both the PAH and NSCLC. We found that the higher expression m7G‐related genes CD163 and SPP1 were related to the immune cells infiltrations and progression in the NSCLC and PAH disease.

Through the GO and KEGG analysis of the DEGs in lung tissues of PAH and NSCLC patients, compared with the NSCLC, the inflammatory response and IL‐17 pathway might play important roles in the PAH. IL‐17 was identified a potential target in the PAH [23]. IL‐17 was mainly produced by Th17, a subset of T cells. It was reported that the higher activation and proliferation of CD4+ T cells was associated with a higher expression of IL‐17 in PAH [24].

Previous studies had reported that in the lung tissue of PAH, the inflammatory cells were present, and the immune cells distribution was changed. It was reported that CD8+T cells were the most important immune cells in the PAH [25]. The CD8+ T cells were highly elevated in the PAH and CD8+T cells were contributed to the development of PAH. CD8+ T cells were accumulated in the remodeled pulmonary vasculature. Also, our study suggested that gammadelta T cells, Tr1 cells, nTreg cells, Th2, and Th17 were elevated in the PAH. Like CD8+T cells, the CD4+T cells are required for the development of the PAH. It was suggested that the T‐lymphocyte subsets, which include helper T cells (Th cells), cytotoxic T cells, and regulatory T cells (Tregs), play different roles in PAH. Among the Th cell subtypes, exploration has shown that Th1 and Th17 cells widely cause the inflammatory and autoimmune responses in PAH by producing interleukin‐6, interleukin‐2, interleukin‐21, interferon‐γ, and tumor necrosis factor‐α [26].

M7G methylation‐mediated various cellular functions. In this study, we identified two m7G‐related genes: CD163 and SPP1. Like cancer, expression of CD163 was significantly related to the CD8+T cells infiltration and macrophages. CD163 was a member of the SRCR family class B expressed on subpopulations of mature tissue macrophages [27]. It was also a marker of antiinflammatory macrophages subtype, M2 macrophage [28]. The high expression of CD163 was mainly induced by antiinflammatory cytokines, including interleukin‐10. CD163+ macrophages were related to the function of the T cells. It was suggested that CD163+CD204+ tumor‐associated macrophages could regulate the function and apoptosis of T cell [29], which suggested that CD163+macrophages influenced the immune regulation and prognosis of the tumor through regulating the T cell function. As shown in various studies, CD163 expression was correlated with worse prognosis of various kind of cancer, including glioma [30] and colorectal cancer [31].

Furthermore, the recruitment of M2 macrophages vital for the development of the PAH [32]. The expression of the CD163 might be used to assess the inflammation of PAH.

SPP1 (secretory phospho‐protein1), also known as osteopontin, is a chemokine‐rich matrix phosphoglycoprotein [33]. SPP1 is produced by various organ, including lung and kidney [34]. SPP1 was a poor prognosis factor of NSCLC [35]. SPP1 consist with the previous studies, NSCLC was present with high CD4+T cells infiltration [36]. SPP1 expression is significantly correlated with the infiltrating CD4+ T cells in NSCLC [37]. SPP1 might induce resistance to the EGFR‐TKI, and influence tumor immune infiltration [35]. The methylations were independent prognostic factors of LUAD [35]. In the NSCLC, SPP1‐CD44 was reported to play a critical role in the cancer chemoresistance and cell communications [34].

SPP1 also played important role in the progression of PAH. Previous studies have found that the SPP1 contribute to the development of PAH via enhancing pulmonary vascular smooth muscle cell (PVSMC) proliferation [33]. Our study also shows that SPP1 plays an important role in the cell interaction of PAH, but the current research is still very limited. Our study suggested that the higher expression of CD163 and SPP1 was related to the disease progression of the PAH and NSCLC. The involvement of macrophages and T cells is the common characteristic of PAH and NSCLC.

### 4.1. Limitation

This study has some limitations. First, the association found in this study cannot infer causation because the sample size was small. Second, the overlapped DEGs were not verified by the experiment. Next, we are going to verify the assumption.

## Ethics Statement

This study was approved by the Ethics Committee for Human Research of the Third Affiliated Hospital of Sun Yat‐sen University. All the participants provided written informed consent.

## Disclosure

All the authors have read and approved the final version of this manuscript.

## Conflicts of Interest

The authors declare that the research was conducted in the absence of any commercial or financial relationships that could be construed as a potential conflict of interest.

## Author Contributions

Yuxia Huang and Sheng Yan recruited the participants and collected samples from patients with PAH. Yuxia Huang collected and assembled the data and edited the manuscript. Jing Zhu and Wentian Zhang revised the manuscript. All authors were involved in the experimental design, data interpretation, and manuscript preparation. Yuxia Huang and Wentian Zhang contributed equally to this work.

## Funding

No funding was received for this manuscript.

## Data Availability

The data generated in this study are publicly available in Gene Expression Omnibus (GEO) at GSE113439, GSE15197, GSE117261, GSE81096, GSE229361, and GSE68465.
